# Allelic Analysis of Sheath Blight Resistance with Association Mapping in Rice

**DOI:** 10.1371/journal.pone.0032703

**Published:** 2012-03-12

**Authors:** Limeng Jia, Wengui Yan, Chengsong Zhu, Hesham A. Agrama, Aaron Jackson, Kathleen Yeater, Xiaobai Li, Bihu Huang, Biaolin Hu, Anna McClung, Dianxing Wu

**Affiliations:** 1 State Key Lab of Rice Biology, Institute of Nuclear-Agriculture Sciences, Zhejiang University, Hangzhou, China; 2 Rice Research and Extension Center, University of Arkansas, Stuttgart, Arkansas, United States of America; 3 Dale Bumpers National Rice Research Center, United States Department of Agriculture-Agricultural Research Service, Stuttgart, Arkansas, United States of America; 4 Department of Agronomy, Kansas State University, Manhattan, Kansas, United States of America; 5 United States Department of Agriculture-Agricultural Research Service, Southern Plains Area, College Station, Texas, United States of America; 6 University of Arkansas at Pine Bluff, Pine Bluff, Arkansas, United States of America; 7 Rice Research Institute, Jiangxi Academy of Agriculture Science, Nanchang, China; University of New England, Australia

## Abstract

Sheath blight (ShB) caused by the soil-borne pathogen *Rhizoctonia solani* is one of the most devastating diseases in rice world-wide. Global attention has focused on examining individual mapping populations for quantitative trait loci (QTLs) for ShB resistance, but to date no study has taken advantage of association mapping to examine hundreds of lines for potentially novel QTLs. Our objective was to identify ShB QTLs via association mapping in rice using 217 sub-core entries from the USDA rice core collection, which were phenotyped with a micro-chamber screening method and genotyped with 155 genome-wide markers. Structure analysis divided the mapping panel into five groups, and model comparison revealed that PCA5 with genomic control was the best model for association mapping of ShB. Ten marker loci on seven chromosomes were significantly associated with response to the ShB pathogen. Among multiple alleles in each identified loci, the allele contributing the greatest effect to ShB resistance was named the putative resistant allele. Among 217 entries, entry GSOR 310389 contained the most putative resistant alleles, eight out of ten. The number of putative resistant alleles presented in an entry was highly and significantly correlated with the decrease of ShB rating (*r* = −0.535) or the increase of ShB resistance. Majority of the resistant entries that contained a large number of the putative resistant alleles belonged to *indica*, which is consistent with a general observation that most ShB resistant accessions are of *indica* origin. These findings demonstrate the potential to improve breeding efficiency by using marker-assisted selection to pyramid putative resistant alleles from various loci in a cultivar for enhanced ShB resistance in rice.

## Introduction

Rice (*Oryza sativa* L.) feeds more than half of the world's population [1] and genetic improvement of this food crop can serve as a major component of sustainable food production. Rice sheath blight (ShB), caused by the soil-borne fungal pathogen *Rhizoctonia solani* Kühn, is a major disease of rice that greatly reduces yield and grain quality worldwide [2]. Due to the high cost of cultural practices and the phytotoxic influence associated with the application of fungicides, the use of ShB resistant cultivars is considered the most economical and environmentally sound strategy in managing this disease. Understandings of genetic control will facilitate cultivar improvement for this disease and secure global food production.

The necrotrophic ShB pathogen has a broad host range and no complete resistance has been identified in either commercial rice cultivars or wild related species [3], [4]. However, substantial differences in susceptibility to ShB among rice cultivars have been observed under field conditions [5], [6]. Differential levels of resistance and the associated resistance genes have been studied among rice germplasm accessions [7]. Rice ShB resistance is believed to be controlled by multiple genes or quantitative trait loci (QTLs) [8]. Since Li et al. [9] first identified ShB QTLs using restricted fragment length polymorphism (RFLP) markers under field conditions, over 30 resistant ShB QTLs have been reported using various mapping populations, such as F_2_s [10]–[14], double haploid (DH) lines [15], recombinant inbred lines (RILs) [8], [16]–[18], near-isogenic introgression lines (NIL) [19] and backcross populations [20]–[23]. ‘Teqing’ and ‘Jasmine 85’ have been repeatedly involved in these studies as the ShB resistant parents. We are the first to map rice ShB QTLs using association mapping strategy in a global germplasm collection.

Association mapping, known as linkage disequilibrium mapping, is a high-resolution method for the dissection of complex genetic traits in plants [24]–[26]. Compared with the traditional bi-parental mapping, association mapping has the benefits of (1) comprehensive mapping resolution with genome-wide scanning; (2) consuming far less time since no parental crosses need to be generated [27], [28]; and (3) potentially more QTLs and alleles can be detected by evaluation of various genomes. Since the successful application of association mapping in humans, researchers have made great advances in utilizing this strategy as a genomics tool in diverse plant species, including Arabidopsis (*Arabidopsis thaliana*) [29]–[31], maize (*Zea mays ssp. mays*) [27], [32]–[34], barley (*Hordeum vulgare* L.) [35], [36], tomato (*Solanum lycopersicum* L.) [37], sweet sorghum [(*Sorghum bicolor* (L.) Moench)] [38], [39], wheat (*Triticum aestivum* L.) [40], and rice [41]–[47].

In association mapping, each identified marker usually has multiple alleles in the mapping panel and each allele in a marker locus contributes differently to the associated trait. Agrama and Yan [42] reported that three alleles at each of three associated loci (allele 87 of RM490, 105 of RM413 and 122 of RM277) and two alleles at another locus (182 and 183 of RM263) had significantly greater contribution to straighthead resistance than other counterparts. Li et al. [47] determined that allele 126 bp had the greatest effect on increasing grain yield, plant weight and grains/panicle branch among eight alleles of RM471. There is no study on allelic distribution for associated loci in a global rice germplasm collection.

Linkage disequilibrium (LD), defined as the non-random association of alleles at separate loci located on the same chromosome [24], is a prerequisite for association mapping. The distance at which LD declines with genetic or physical distance determines the marker density needed for achieving a reasonable mapping resolution. The extent of LD may vary among different genomic regions [48]. Numerous studies on global germplasm collections indicate 25 cM as a reasonable resolution for association mapping in rice [41], [47], [49]. In our study, we used 154 simple sequence repeat (SSR) markers plus an indel to provide coverage of 10 cM across the rice genome for sufficient mapping resolution.

Accurate phenotyping is essential for mapping, especially when the target trait is controlled by multiple genes or QTLs such as ShB resistance. All the previous studies phenotyped ShB resistance under field conditions with only one exception, Liu et al. (2009) [18], where a micro-chamber method (MCM) was adapted. The MCM described by Jia et al. (2007) [6] has proven to effectively minimize the confounding effects of environmental and morphological factors, thus generating more reliable data. Furthermore, numeric measurement of ShB in the MCM should be more accurate than the traditional visual scoring under field conditions. Because of these advantages, the MCM has been widely applied in studies of ShB resistance [18], [50], [51].

Using association mapping, our objectives were to 1) map QTLs associated with ShB resistance phenotyped with the MCM, 2) identify putative resistant alleles in a global germplasm collection, and 3) explore the use of ShB putative resistant alleles in a breeding program.

## Results

### Variation of ShB severity ratings

**Figure 1:**
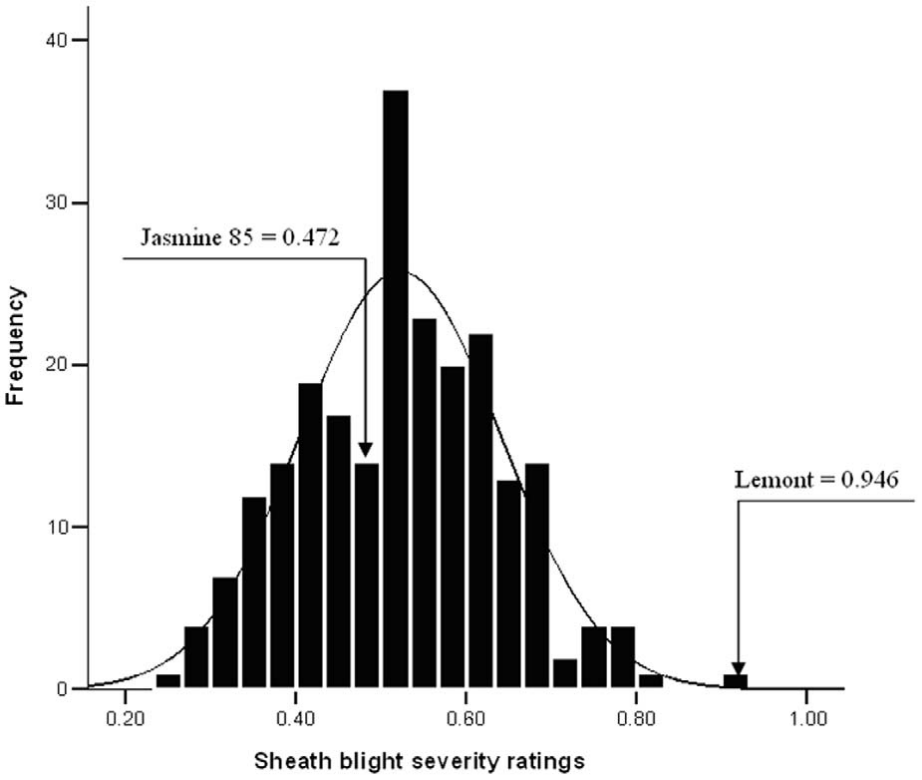
Distribution of sheath blight severity ratings among 217 sub-core entries. The rating was averaged over 18 plants, three in each of six replications using the micro-chamber method with the resistant check Jasmine 85 and susceptible Lemont.

The 217 sub-core entries in the mapping panel originated from fifteen geographic regions including 77 countries worldwide. India had the most entries (6.5%), followed by China (5.5%), Indonesia (4.1%), Japan (4.1%) and Taiwan (4.1%). Their name, origin, ShB severity rating, structure group and entry number in the Genetic Stocks Oryza (GSOR) collection (http://www.ars.usda.gov/Main/docs.htm?docid=8318) are presented in (Table S1). The ShB severity ratings among the 217 entries were distributed normally, ranging from 0.256±0.111 to 0.909±0.096 with an average of 0.521±0.008 (Fig. 1). The resistant check Jasmine 85 was rated 0.472±0.021 and susceptible check Lemont was rated 0.946±0.080. Twenty-four entries (11.1%) were significantly more resistant to ShB than Jasmine 85 at the 5% level of probability while 54 others (24.9%) had similar resistance.

### Population structure

**Figure 2:**
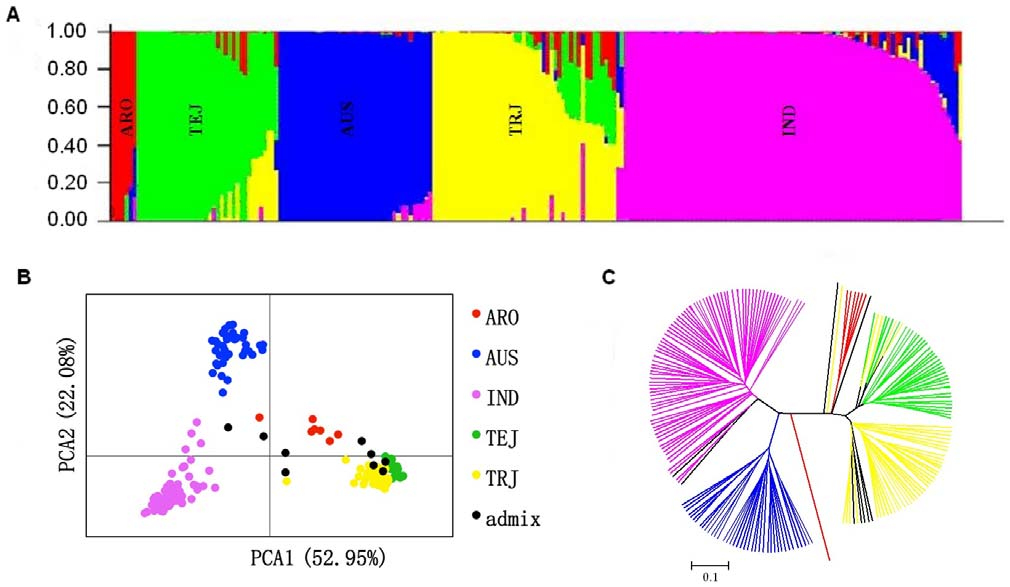
The structure analysis divided our population into five groups, which was validated by the principal components analysis (PCA) and cluster analysis. (A) Population structure analysis of 217 sub-core entries showing five sub-groups, the estimated membership probability listed on the y-axis and each entry represented by a thin vertical line in different color: red = ARO, *aromatic*; blue = AUS, *aus*; pink = IND, *indica*; green = TEJ, *temperate japonica* and yellow = TRJ, *tropical japonica*. (B) The spatial distribution of the entries with two dimensions in the principal components analysis (PCA). (C) The unweighted pair-group method with arithmetic mean (UPGMA) tree based on Nei's genetic distance using five sub-group partitioning.

Structure analysis from Q1 to Q10 across twenty runs for the 217 sub-core entries genotyped with 155 genome-wide DNA markers using STRUCTURE demonstrated that when Q reached five, the Pr(Q) became more-or-less plateaued, so Q5 captured the major structure in our data. Thus, the mapping panel was divided into five subgroups and each entry was classified to an appropriate subgroup using STRUCTURE. Inferred by reference cultivars recommended by Agrama et al. [52], [53], the five subgroups were denoted as *temperate japonica* (TEJ), *aus* (AUS), *aromatic* (ARO), *indica* (IND), and *tropical japonica* (TRJ) (Fig. 2A). A similar structural pattern was seen with the PCA analysis with the first two axes explaining 75.03% of variation (Fig. 2B). Furthermore, the genetic distance based on cluster analysis also divided the mapping panel into five major clusters (Fig. 2C). All three approaches led to the same conclusion: a five-group structure could clearly and sufficiently explain the existing genetic diversity in the mapping panel. In the mapping panel, IND had the most entries (86), followed by TRJ (49), AUS (39), TEJ (36), and ARO (7). Among 24 entries having greater resistance to ShB than the resistant check, Jasmine 85, 20 belonged to IND, two to AUS and one each to TRJ and admix (TRJ-AUS-IND).

### Determination of the best fit model

**Figure 3:**
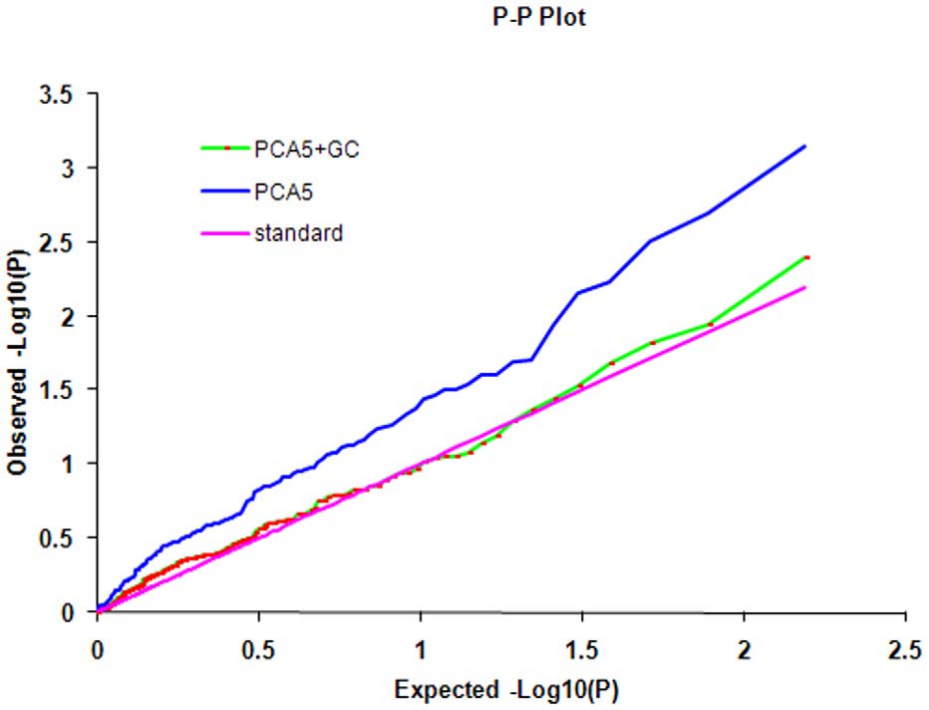
The cumulative distributions of observed -Log10(P) values before and after genomic control (GC) in PCA5 model. The genomic controlled PCA5 (PCA5+GC) model had a more uniform distribution and closer to the expected -Log10(P) values, thus greater power to control the Type I errors than PCA5.

From dimension 1 to 10 in the PCA and structure Q1 to Q10, PCA5 had the smallest Bayesian Information Criterion (BIC) value, indicating that PCA5 should be the best fit model to map ShB QTLs (Table 1). Hence, we tested each of 155 molecular markers for association with ShB resistance using PCA5, and plotted the observed versus expected -Log10(P) before and after correction using the genomic control (GC). The plots of the PCA5+GC were distributed more uniformly and was much closer to the expected -Log10(P) than PCA5 alone (Fig. 3). In other words, the PCA5+GC model showed better control for Type I errors. Therefore, the GC approach was applied to correct the biased estimation. The P values generated from the PCA5 model after the GC correction were used to present the significance level of each marker.

**Table 1:** Comparative analysis of different subgroups using structure (Q model) and different dimensions in principal components analysis (PCA) for association mapping of sheath blight resistance using 217 entries genotyped with 155 molecular markers.

Model	Deviance	BIC	Model	Deviance	BIC
Simple model	−303.4	−292.6	PCA1	−368.6	−352.5
Q2	−361	−355.6	PCA2	−370.4	−348.9
Q3	−372.7	−345.8	PCA3	−373.8	−346.9
Q4	−377.1	−344.8	PCA4	−388.4	−356.1
Q5	−376.1	−338.4	PCA5	−399.5	−361.9
Q6	−376.5	−333.5	PCA6	−403.7	−360.7
Q7	−393.9	−345.5	PCA7	−403	−354.6
Q8	−383.1	−329.3	PCA8	−404.5	−350.7
Q9	−396.7	−337.6	PCA9	−407.1	−347.9
Q10	−389	−324.4	PCA10	−407.8	−343.2

BIC: Bayesian Information Criterion (the smaller, the better); Deviance: −2log likelihood.

### Marker loci and their alleles associated with sheath blight

**Figure 4:**
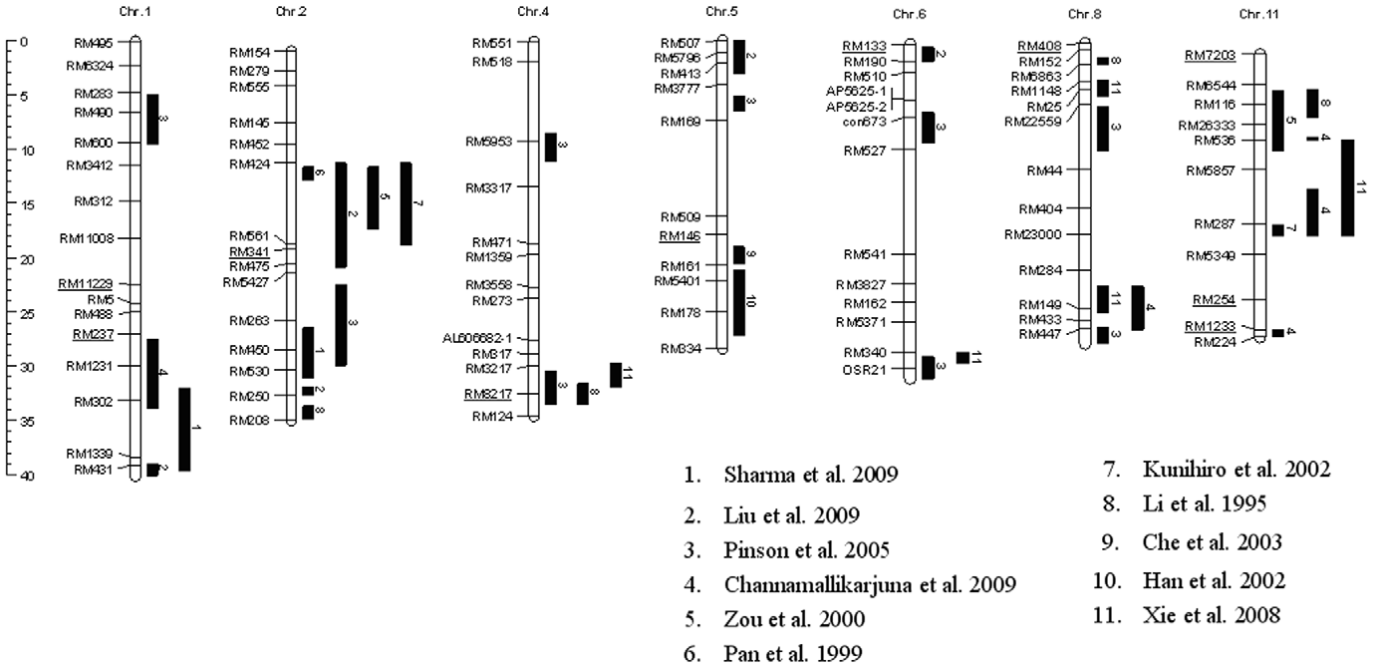
The physical position of marker loci significantly associated with sheath blight in our study (with underlines) in comparison with previous studies. The black bars show the estimated location according to their flanking markers. The positions of marker loci were cited from the Annotated Nipponbare Sequence 2009 on Gramene (http://www.gramene.org/).

Ten marker loci were identified to be significantly associated with ShB resistance at the probability level of 5% or lower, three on chromosome (Chr) 11, two on Chr1, and one each on Chr2, 4, 5, 6 and 8 (Table 2, Fig. 4). RM237 on Chr1 at 27.1 Mb had the highest significance rating for ShB at the 0.002 level of probability. RM11229 on the long arm of Chr1 explained the most phenotypic variation (9.5%) with significance at the 0.044 level of probability. RM11229 and 1233 each had six alleles, the most among the 217 sub-core entries, followed by RM341 and 254 (five alleles), RM237, 8217,146 and 408 (four), RM133 (three) and RM7203 (two) (Table 2).

**Table 2:** Marker loci significantly associated with sheath blight resistance, their physical locations on chromosomes (Chr), allele size in 217 entries, number of entries with the allele, and their mean sheath blight (ShB) rating.

Marker	Chr.	Position (Mb)	P value	Rsq_Marker^a^	Allele (bp)	Number of Entries	ShB Mean^b^
RM11229	1	22.6	0.044	9.5%	158*	18	0.414
					192	21	0.515
					195	21	0.473
					198	13	0.532
					207	14	0.608
					224	12	0.466
RM237	1	27.1	0.002	6.9%	122	19	0.526
					128*	32	0.473
					130	105	0.515
					132	20	0.635
RM341	2	19.3	0.041	4.1%	135	89	0.558
					138	39	0.545
					139*	17	0.447
					141	15	0.579
					171	39	0.461
RM8217	4	32.6	0.044	3.2%	178	67	0.581
					182	19	0.534
					184	65	0.482
					186*	49	0.476
RM146	5	18	0.021	3.8%	330	26	0.539
					332	127	0.512
					340*	28	0.463
					344	28	0.591
RM133	6	0.2	0.043	2.4%	228	89	0.557
					230*	104	0.479
					232	22	0.577
RM408	8	0.1	0.023	4.0%	117	18	0.577
					119*	105	0.478
					125	18	0.498
					127	57	0.591
RM7203	11	1.1	0.033	1.9%	88*	120	0.470
					104	80	0.589
RM254	11	23.7	0.030	5.3%	159	25	0.580
					161	36	0.564
					163	54	0.511
					167	50	0.480
					169*	12	0.463
RM1233	11	26.5	0.036	5.1%	158	97	0.538
					164	12	0.543
					168	15	0.593
					173	12	0.520
					177*	35	0.451
					179	12	0.524
Resistant check ‘Jasmine 85’							0.472

a Rsq_Marker - total explained phenotypic variation.

b The mean of ShB severity rating for the entries with the allele.

Allele*: Putative resistant allele that had the lowest ShB mean at the marker locus.

Among the six alleles of RM11229, allele 158 was present in 18 entries that had the lowest average ShB rating (0.414), and thus, it was designated as the ‘putative resistant allele’ of this marker locus. Accordingly, ten alleles, one each from the ten associated marker loci, were noted as the putative resistant allele in Table 2 because they had the greatest effect to decrease ShB among all the alleles for their respective loci (Table 2). ShB rating was the smallest for putative resistant allele 158 of RM11229 among the ten putative resistant alleles. Of the other five putative resistant alleles, 139 of RM341 (present in 17 entries), 340 of RM146 (28 entries), 88 of RM7203 (120 entries), 169 of RM254 (12 entries) and 177 of RM1233 (35 entries), had lower ShB means ranging 0.447–0.470 than the resistant check Jasmine 85 (0.472), suggesting a stronger effect for resistance to ShB than Jasmine 85. The remaining four putative resistant alleles had similar ShB ratings with Jasmine 85, suggesting a similar effect for the level of ShB control.

Among the ten putative resistant alleles, allele 88 of RM7203 was the most prevalent and existed in 120 (55%) of 217 entries in the mapping panel, followed by allele 230 of RM133 and 119 of RM408 (48% of the lines), allele 186 of RM8217 (23%), allele 340 of RM146, 128 of RM237 and 177 of RM1233 (13–16%), allele 139 of RM341 and 158 of RM1229 (8%), and allele 169 of RM254 (6%).

### Number of putative resistant alleles and sheath blight resistance

As the number of putative resistant alleles in the germplasm increased, so did germplasm resistance to ShB (Table S1). GSOR 310389 from Korea contained the most putative resistant alleles, eight out of ten, and had a ShB rating of 0.351 which was significantly more resistant than the resistant check Jasmine 85 which contained three putative resistant alleles and had a ShB rating of 0.472. Among seven entries containing six putative resistant alleles with a mean of 0.386 ShB, GSOR 310475 and 311475 were more resistant than Jasmine 85 and had ShB ratings of 0.324 and 0.336, respectively. Among 28 entries having five putative resistant alleles with a mean ShB rating of 0.444, seven were significantly more resistant than Jasmine 85. Seven, out of 35 entries which carried four putative resistant alleles and had a mean ShB 0.466, were identified to be significantly more resistant than Jasmine 85. The mean ShB ratings for entries containing three, two, one and zero putative resistant alleles were 0.483, 0.535, 0.582 and 0.598, respectively. There was a strong and negative correlation between the ShB severity rating and number of putative resistant alleles (*r* = −0.535, *p*<0.0001).

**Figure 5:**
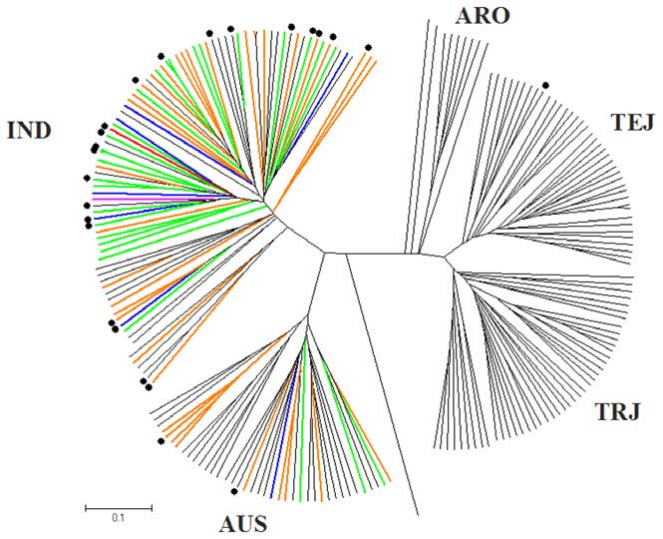
UPGMA tree based on Nei genetic distance for 217 sub-core entries. Twenty four entries marked with • were significantly more resistant to sheath blight than the resistant check ‘Jasmine 85’. The number of ‘putative resistant alleles’ present is distinguished by branch color: Red = eight putative resistant alleles, Pink = seven, Blue = six, Green = five, and Orange = four.

Our mapping results showed that most entries containing a large number of putative resistant alleles were IND (Fig. 5 and Table S1). All entries with six or more putative resistant alleles were IND with only one exception of AUS. Among 28 entries with five putative resistant alleles, 25 were IND and the remaining three were AUS. There were 35 entries with four putative resistant alleles, nine were AUS, one was an admix of TRJ, AUS and IND, and the remaining 25 were IND. Among 35 entries with three putative resistant alleles, 18 were IND, eight AUS, seven TRJ and two admixes of IND. However, among 51 entries without a single putative resistant allele, 26 were TEJ, 18 TRJ, four ARO and two admixes of TRJ-TEJ-ARO, and one IND. Among 72 entries that carried four or more putative resistant alleles, 58 (81%) were IND and 13 AUS (18%) plus an admix of TRJ-AUS-IND.

## Discussion

### Pyramiding putative resistant alleles for cultivar improvement

*R. solani* is a soil-borne necrotrophic fungus and its group AG1-IA has a broad host range including rice, maize, wheat, sorghum, bean (*Phaseolus* spp.) and soybean [*Glycine max* (L.) Merr.] [54]. The pathogen's ability to persist in soil and on crop residues allows it to, survive in multiple ways and makes disease management difficult. There is no complete resistance to ShB in rice because the resistance is quantitatively controlled by numerous genes or quantitative trait loci (QTLs). More than 30 QTLs responsible for ShB have been reported in rice [8], [10]–[23]. However, all of these studies have been limited to conventional mapping populations from a small number of parents, which limits the alleles in the progeny to those present in the parental lines.

From our diverse mapping panel including 217 sub-core entries, we identified ten marker loci significantly associated with ShB resistance, each locus had numerous alleles, each allele contributed differently to ShB resistance, and the putative resistant allele of each locus contributed the most (Table 2). Highly significant correlation demonstrated that as more putative resistant alleles pyramided in a germplasm entry, the entry had greater resistance to ShB. Among 24 entries that were significantly more resistant than the resistant check Jasmine 85 that had three putative resistant alleles, 17 (71%) contained four or more putative resistant alleles. Among 54 entries that had similar resistance with Jasmine 85, 29 (54%) possessed three or more putative resistant alleles.

These findings suggest that marker-assisted breeding for ShB resistance can be conducted on an allelic level by pyramiding putative resistant alleles in a cultivar. This can be accomplished in a two pronged approach by combining parental lines based upon their combination of number of different ShB resistant loci and possessing the putative resistant alleles at these loci. For example, resistant entry GSOR 310389 that has eight putative resistant alleles could be crossed with a commercial cultivar having fewer putative resistant alleles, and the progeny would be selected based on those having the most highly putative resistant alleles at the most loci. The breeding program would end up with the selection of progeny containing the most putative resistant alleles, potentially having greater resistance to ShB than either parental line.

Pyramiding responsible genes has been successfully applied in rice breeding for disease resistance including bacterial blight (*Xanthomonas oryzae* pv.) and blast (*Magnaporthe oryzae*). Singh et al. (2001) [55] pyramided three bacterial blight resistance genes, *xa5*, *xa3* and *Xa21* into a cultivar PR106 and increased resistant spectrum and level to the disease pathogen. Hittalmani et al. (2000) [56] gathered three blast resistant genes, *Pi1*, *Piz5* and *Pita* in a cultivar Co39 using RFLP and PCR based marker technology and the improved cultivar demonstrated a durable resistance to multiple biotypes of blast pathogen. Similarly, McClung et al. (1997) [57] combined three major genes, *Pi-d*, *Pi-z* and *Pi-k^h^*, and bred blast resistant cultivar Jefferson. All the successful applications of gene pyramiding have been at the gene loci level. Our findings in this study will help enhance the application to allelic level in crop breeding. The allelic application will improve breeding efficiency, increase cultivar resistance to sheath blight in rice and ultimately secure food production worldwide.

Allelic analysis can only be applied in association mapping where large number of diversified genotypes are used and multiple alleles are involved at each associated marker locus in the mapping panel. Using this method, germplasm accessions that are identified in the association mapping strategy to possess multiple putative resistant alleles can be crossed with other accessions that have a different complement of putative resistant alleles. The selection of progeny possessing the most putative resistant alleles should be more effective than it is for resistant loci. In this regard, association mapping offers advantages for identifying parental material and specific alleles that can enhance breeding.

### Putative resistant alleles and ancestry background for sheath blight

Jia et al. [58] reported 52 entries that are significantly more resistant to ShB than Jasmine 85. The resistant entries were identified from 1,794 entries of the USDA rice core collection that has 35% *indica*, 27% *temperate japonica*, 24% *tropical japonica*, 10% *aus* and 4% *aromatic* genotypes [52]. Based on the ancestry classification, there are 621 *indica* entries in the core and 45 of them are included in the resistant list, making a resistance frequency of 7.2% for *indica* germplasm. Accordingly, the resistance frequency is 2.8% for *aromatic*, 1.7% for *aus*, and 0.2% each for *temperate japonica* and *tropical japonica*. In a study conducted by Zuo et al. [23], *japonica* cultivars showed higher sheath blight severity than *indica* cultivars. They describe a general observation that *japonica* rice is more susceptible than *indica* rice. Furthermore, Jasmine 85, Tetep and Teqing, used as parents in many studies on mapping ShB resistance, all belong to *indica*.

This study demonstrated that: 1) a majority of the ShB putative resistant alleles existed in *indica* germplasm, 2) most of the resistant entries with a large number of putative resistant alleles were *indica*, conversely 3) only a very small portion of putative resistant alleles existed in *japonica*, and 4) the most susceptible entries with very few or no putative resistant alleles were *japonica* (Fig. 5 and Table S1). Entry GSOR 310389 is an example which had eight out of ten putative resistant alleles, showed a high level of resistance to ShB, and is *indica*. The results from association mapping match well with the phenotypic observation that most resistant genotypes are *indica* and resistant germplasm is rare in *japonica*.

### ShB associated markers and QTL identification

Our genome-wide search found ten marker loci that were significantly associated with sheath blight resistance (Fig. 4). Both RM11229 (Chr1 at 22.7 Mb) and RM7203 (Chr11 at 1.1 Mb) are novel QTLs that have not been previously reported. RM11229 is approximately 5.0 Mb away from a ShB QTL reported by Channamallikarjuna et al. [16] and RM7203 resides about 3.3 Mb away from one QTL identified by Li et al. [9]. The remaining eight QTLs identified in this study were either quite near (less than 1.4 Mb distant) or within the interval of previously identified QTLs. The ten associated markers identified in this study were located on seven chromosomes (Chr1, 2, 4, 5, 6, 8 and 11) (Fig. 4).

On Chr1 our study identified RM11229 and RM 237, which occur within 4.4 Mb of each other, as markers associated with ShB resistance. RM11229 explained the most phenotypic variation (9.5%) and its putative resistant allele 158 bp had the smallest average ShB score (0.414 in Table 2), indicating the greatest resistance among the ten putative resistant alleles. RM237 was the most significant marker for ShB resistance (*p* = 0.002). Therefore, the 4.4 Mb gap between RM11229 and RM237 on Chr1 should be a target area for fine-mapping ShB resistant genes in rice. RM237 at 26.8 Mb is near the ShB QTL region spanning 27.6 to 34 Mb found by Charnnamallikarjuna et al. [16].

On Chr2 the identified marker RM341 located at 19.3 Mb, overlapped with the *qShB2-1* (11.4∼21.5 Mb) reported by Liu et al. [18] and was near the *qSB-2* (11.8∼17.5 Mb) by Zou et al. [14], and *qSBR-2* (11.4∼19.0 Mb) by Kunihiro et al. [15]. RM341 explained 4.1% of phenotypic variation and its putative resistant allele sized 139 bp existed in seventeen entries that were more resistant to ShB than Jasmine 85 on average.

On Chr4, three reports uniformly indicated ShB QTLs on the long arm at 29.8 to 33.6 Mb. This is a very narrow region of 3.8 Mb on the physical map, and corresponds to a small estimated cM distance in the mapping population developed from susceptible Lemont and resistant Teqing parents [8], [9], [59]. Our identified marker RM8217 at 32.6 Mb was within the *qSB-4-2* (30.6∼33.6 Mb) by Pinson et al. [8] and *Qsbr4a* (31.7∼33.6 Mb) by Li et al. [9], and in close proximity (within 0.6 Mb) to *QRlh4* (29.8∼32.0 Mb) by Xie et al. [59]. This small area confirmed by multiple studies strongly suggests a reliable location harboring ShB QTL.

On Chr5, 6 and 8, we identified one ShB associated marker from each chromosome. RM146 on Chr5 at 18.1 Mb is close to the *Rsb 1* reported by Che et al. [10] between RM164_320_ at 19.1 Mb and RM39_300_ at 20.7 Mb near the centromere. RM133 (Chr6 at 0.2 Mb) and RM408 (Chr8 at 0.1 Mb) were close to the *qShB6* (0.5∼1.8 Mb) described by Liu et al. [18] and the *QSbr8a* (1.5∼2.1 Mp) by Li et al. [9], respectively. The putative resistant alleles of both RM133 (allele 230 bp) and RM408 (allele 119 bp) were common with more than 45% of entries among the 217 sub-core entries in the mapping panel.

RM7203 on the short arm of Chr11 at 1.1 Mb was a novel ShB QTL that has not been reported. The putative resistant allele 88 of RM7203 existed in 55% of the 217 entries, so was the most common allele and had a ShB mean (0.470) similar to Jasmine 85 (0.472).

At the bottom of Chr11, two markers RM254 (at 23.7 Mb) and RM1233 (at 26.5 Mb) were identified, explaining relatively high phenotypic variation among ten markers, 5.3% and 5.1%, respectively. The RM1233 was one of the flanking markers for qSBR11-1 (26.5∼27.2 Mb) reported by Channamallikarjuna et al. [16]. The putative resistant alleles, 169 bp of RM254 and 177 bp of RM1233, were in 12 and 35 entries, respectively. Their ShB ratings were lower than Jasmine 85 in average, indicating a stronger resistance.

Above comparisons confirm eight out of ten marker loci identified in our association mapping with two novel QTLs, RM11229 and RM7203, for sheath blight resistance. The confirmation of previously identified QTLs provides validation for the accuracy of QTLs identified in our study. Furthermore, the comparisons demonstrate that association mapping can locate many QTLs over the entire genome since the mapping panel includes a large number of diversified entries of germplasm. In biparental linkage mapping studies, fewer QTLs are typically identified and can only be located in a limited area in the genome where the two parents differ.

## Materials and Methods

### Germplasm panel

The rice mini-core collection of United States Department of Agriculture (USDA) contains 217 entries [53] derived from 1,794 entries of a core collection [60]. The core collection has been shown to be representative of the genetic diversity found in more than 18,000 accessions of the USDA rice whole collection [60]. The mini-core has proven to be an efficient platform for association mapping and has been successfully applied to mapping QTLs for improving grain yield [47]. We excluded fourteen entries of wild species to minimize interference due to different genetic structure [61] and replaced them with fourteen core entries known to have greater resistance to ShB than Jasmine 85, a common resistant check in the comprehensive evaluation of the core collection [58]. The replacement aimed to enhance detection of QTLs by increasing the frequency of putative resistant alleles in the panel.

### Phenotyping

A complete set of 1,794 entries in the USDA rice core collection was evaluated in 2008, using the MCM with three replications, three plants in each replication following a randomized incomplete block design over time [58]. Rice cultivars, Lemont (susceptible) and Jasmine 85 (resistant), were included as repeated checks in each replicate to serve as standards for evaluation. Both the check cultivars have been used as standard checks in many other studies regarding ShB resistance [11], [12], [14], [18]. In 2009, the 217 entries of the mini-core collection, plus those core entries that showed significantly more resistance than Jasmine 85, were re-evaluated using the same protocol. LSmean of ShB severity from six replications including 18 observations of each entry was used for association mapping.

The isolate RR0140-1 of *R. solani* was selected from 102 isolates collected state-wide from Arkansas rice fields due to its slow growing phenotype [51]. Slow growing isolates cause relatively consistent disease reaction and differentiate susceptible cultivars from moderately resistant ones better than fast growing isolates [51]. Field evaluations showed no differences in disease reactions between the slow growing isolates and the fast ones [51]. Further, the RR0140-1 isolate have been adapted by numerous studies [6], [18], [50]. Pathogen inoculum of RR0140-1 were grown by placing sclerotia in the centre of potato dextrose agar (PDA) plates (Sigma-Aldrich, St. Louis) containing 0.005% (wt/vol) tetracycline, and then transferred to a fresh PDA medium for 5–6 days at 27°C under darkness. Mycelium discs (7 mm in diameter) were excised from the outer growing area in the culture plate where the outer mycelia were mostly active. Rice seedlings were inoculated at the three-leaf stage.

In the greenhouse, each 12×12 cm pot was filled with pre-sterilized soil to ensure that the study was not confounded by the presence of soil borne *R. solani* inoculum. Pots with drainage holes were placed in flats filled with shallow water (∼5 cm). Five seeds of each accession were planted in each pot and thinned to three uniform plants before pathogen inoculation. The three remaining plants in a given pot were referred to as one experimental unit or replicate. Each of the three seedlings in a pot was individually inoculated with a round mycelium disc of RR0140-1 pathogen as described by Jia et al. [6] with modification. Each disk was pressed up to the base of the seedling stem, assuring that the mycelium was in contact with the plant. After inoculation, each pot was immediately covered with a 2-liter soft drink bottle with the bottom and cap removed. Relative humidity was maintained over 80% in the bottle, which favoured growth of the sheath blight pathogen on the plants. The greenhouse temperatures were set for day/night at 30/22°C, respectively with a 12 h photoperiod.

Plant response to the sheath blight pathogen was measured using the ratio between the height of the pathogen growing up the plant and the height of the leaf collar on the last emerged leaf. Because mature plant height varied from 70 to 202 cm in this collection [60], the ratio excluded possible interference of plant height in scoring disease response. Therefore, the smaller the ratio, the greater the resistance was for an entry. Measurements were taken when the ratio reached 1.0 for 75% of the susceptible check plants, Lemont, so that the maximum susceptibility was scored 1.0.

ShB rating data were analyzed using the GLIMMIX procedure in SAS version 9.1.3 [62]. The experimental design of randomized incomplete block formed the basis of the statistical model, where the accession is a fixed effect and block is treated as random effect. The LSMEANS option was used to calculate the least-square means (LSMs) of each entry and the LSMs were used for the association mapping. The statistical differences of the accession to each check (Jasmine 85 and Lemont) were determined by a Dunnett's multiple comparison test, using the diff = control option.

### Genotyping

DNA was extracted from leaf tissue of five plants for each of the 217 entries using a rapid alkali extraction procedure [63] and genotyped with 154 SSR markers plus an indel. The 155 molecular markers covered the entire rice genome with an average genetic distance of 10 cM, described by Li et al. [47]. PCR amplifications were performed according to Agrama et al. (2007) [42]. For each marker, forward primers were labeled with either 6FAM, NED or Hex (Applied Biosystems, Foster City, CA, USA or Integrated DNA Technologies, Coralville, IA, USA). The amplifications were performed using MJ Research Tetrad thermal cyclers (Bio-Rad, Hercules, CA, USA). PCR products were pooled based on color and size range of amplified fragments (typically three markers per run along with ROX-labeled size standard), and the DNA was denatured by heating samples at 94°C for 5 min. The samples were separated on an ABI Prism 3730 DNA Analyzer according to the manufacturer's instructions (Applied Biosystems). Data were analyzed using GeneMapper v. 3.7 software (Applied Biosystems).

### Population Structure Analysis

Analysis of population structure in the mapping panel was performed using STRUCTURE software [64], [65]. Rare alleles, with frequency of less than 5% in the panel, were treated as missing data for structure analysis, principal components analysis (PCA), cluster analysis and association mapping. We implemented a model-based clustering method for inferring population structure using distinctive allele frequencies and assigning individuals into Q clusters. Twenty independent runs were performed for each value of Q, ranging from one to ten, using the admixture model with a burn-in of 50,000 iterations followed by 100,000 iterations during analysis. Subgroups were determined on the basis of the following criteria: (1) likelihood plot of these models; (2) stability of grouping patterns across twenty runs; and (3) germplasm information about the materials under study. To validate the population structure and compare the different models, PCA was conducted to obtain eigenvectors for further model testing and association analysis. Genetic distance was calculated with PowerMarker [66] using Nei's method [67]. The resulting unweighted pair-group method with arithmetic mean (UPGMA) tree was viewed using MEGA 4.0 [68].

### Model comparisons and association analysis

The flexible mixed model [69] was used to control population structure. For the purpose of model comparisons, the phenotypic vector is modeled as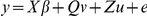, where β is vector of marker effects to be estimated. The term *Qv* contains the coordinates of the individuals of *p* dimensions in Q matrix generated by STRUCTURE [64], [65] and PCA matrix by NTSYSpc version 2.1 [70]; X and Z are the incidence matrices of 1 s and 0 s that relate y to β and 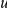, respectively. 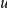 is a vector of polygene background effects; and e is a vector of residual. The phenotypic covariance matrix was assumed to have the form 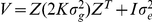, where *K* is the K matrix including relative kinship coefficients defining the degree of genetic covariance between a pair of individuals [71], *I* is an *n*×*n* identity matrix, 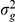 is the genetic variance attributable to genome wide effects, and 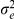 is the residual variance.

When model K or Q+K or PCA+K were tested for a fit, we found little convergence because of the low level of relatedness among entries in the panel. Thus, a simple model (ignoring the effect of population structure), possible linear models of Q2-Q10 (considering different number of subgroups from two to ten) and PCA1-PCA10 (PCA matrix with different number of dimensions from one to ten) were compared for the best fit to ShB determined by Bayesian information criterion (BIC). In order to control false positive rates, the genomic control (GC) method [72] was further used for correcting population structure. The association mapping was conducted using the best fit model with TASSEL v.2.1 [73], followed by the GC. The associated markers with ShB resistance were claimed at the probability level of 0.05. The ShB severity ratings of germplasm entries that carried the same allele in an associated marker locus were averaged to estimate allelic effect on the ShB rating. Among the alleles of each associated marker locus, the allele with the lowest ShB mean was indicative of the strongest effect and was designated as the ‘putative resistant allele’.

## Supporting Information

Table S1 **Accession number in the Genetic Stocks Oryza (GSOR) collection, sheath blight (ShB) mean, cultivar name, country of origin, structural group, and number of putative resistant alleles present for 217 entries from the USDA rice core collection.**
(DOC)
