# Supplementary material for: Allelic Analysis of Sheath Blight Resistance with Association Mapping in Rice
Source: PLoS One. 2012 Mar 12;7(3):e32703. doi: 10.1371/journal.pone.0032703 (PMC3299681; doi:10.1371/journal.pone.0032703)
Supplement: Table S1 — Accession number in the Genetic Stocks Oryza (GSOR) collection, sheath blight (ShB) mean, cultivar name, country of origin, structural group, and number of putative resistant alleles present for 217 entries from the USDA rice core collection. (DOC) [file pone.0032703.s001.doc]

Table S1 Accession number in the Genetic Stocks Oryza (GSOR) collection, sheath blight (ShB) mean, cultivar name, country of origin, structural group, and number of putative resistant alleles present for 217 entries from the USDA rice core collection.

| GSOR | ShB mean | Cultivar name | Country of origin | Structural group* | Putative resistant alleles |
| --- | --- | --- | --- | --- | --- |
|
| 310389 | 0.351 | Won Son Zo No. 11 | Korea | IND | 8 |
| 310791 | 0.499 | Kin Shan Zim | China | IND | 7 |
| 310475 | 0.324 | K8C-263-3 | Suriname | IND | 6 |
| 311475 | 0.336 | Tie 90-1 | China | IND | 6 |
| 310714 | 0.370 | Tchampa | Iran | AUS | 6 |
| 310515 | 0.383 | Nam Dawk Mai | Thailand | IND | 6 |
| 311781 | 0.391 | Krachek Chap | Indochina | IND | 6 |
| 310489 | 0.427 | Chun 118-33 | China | IND | 6 |
| 311466 | 0.467 | Kechengnuo No. 4 | China | IND | 6 |
| 310849 | 0.256 | Pan Ju | China | IND | 5 |
| 311464 | 0.310 | Tiejing No.4 | China | IND | 5 |
| 311187 | 0.311 | Cristal de Angola | Brazil | IND | 5 |
| 311117 | 0.323 | Tranoeup Beykher | Cambodia | IND | 5 |
| 311073 | 0.341 | Tukan Tuna | Indonesia | IND | 5 |
| 311151 | 0.345 | TD 70 | Thailand | IND | 5 |
| 311303 | 0.362 | SL 22-642 | Sierra Leone | IND | 5 |
| 310100 | 0.371 | Ao Chiu 2 Hao | China | IND | 5 |
| 311725 | 0.372 | A 36-3 | Myanmar | IND | 5 |
| 311739 | 0.374 | Fujisaka 5 | Japan | IND | 5 |
| 311748 | 0.377 | Bogarigbeli | Burkina Faso | IND | 5 |
| 310788 | 0.409 | Toga | India | IND | 5 |
| 311582 | 0.411 | Banjul | Gambia | IND | 5 |
| 310015 | 0.414 | Mayang Khang | Indonesia | IND | 5 |
| 311249 | 0.437 | Tono Brea 439 | Dominican Republic | IND | 5 |
| 311554 | 0.474 | Srav Prapay | Cambodia | IND | 5 |
| 310687 | 0.489 | IR 9660-48-1-1-2 | Philippines | IND | 5 |
| 310757 | 0.494 | RP2151-173-1-8 | India | IND | 5 |
| 311278 | 0.499 | Montakcl | Egypt | AUS | 5 |
| 311741 | 0.502 | Soc Nau | Vietnam | IND | 5 |
| 311483 | 0.507 | 4484 | China | IND | 5 |
| 311105 | 0.533 | Hsin Hsing Pai Ku | Taiwan | IND | 5 |
| 310773 | 0.559 | ECIA76-S89-1 | Cuba | IND | 5 |
| 310703 | 0.563 | N-2703 | Nepal | AUS | 5 |
| 311286 | 0.564 | UZ Ros 59 | Uzbekistan | IND | 5 |
| 311491 | 0.575 | You-I B | China | IND | 5 |
| 311046 | 0.585 | IARI 6621 | India | AUS | 5 |
| 311417 | 0.688 | CNTLR80076-44-1-1-1 | Thailand | IND | 5 |
| 311372 | 0.285 | P 3084F4-56-2-2 | Colombia | IND | 4 |
| 311175 | 0.288 | Tsilaitranakoho 706 | Madagascar | TRJ-AUS-IND | 4 |
| 311727 | 0.309 | Nahng Sawn | Thailand | IND | 4 |
| 311746 | 0.316 | Hung Tau Keng | China | IND | 4 |
| 311726 | 0.331 | Pah Leuaud 111 | Thailand | IND | 4 |
| 311613 | 0.362 | Spin Mere | Afghanistan | AUS | 4 |
| 310417 | 0.362 | Bau 157 | Vietnam | IND | 4 |
| 310134 | 0.376 | Berlin | Costa Rica | IND | 4 |
| 311744 | 0.382 | Heo Trang | Vietnam | IND | 4 |
| 310471 | 0.386 | Patnai 6 | Myanmar | AUS | 4 |
| 310442 | 0.386 | PD 46 | Sri Lanka | IND | 4 |
| 311193 | 0.388 | Java Long Grain | Indonesia | AUS | 4 |
| 310420 | 0.394 | Thang 10 | Vietnam | IND | 4 |
| 310846 | 0.403 | Kao Chio Lin Chou | Taiwan | IND | 4 |
| 311684 | 0.426 | Hi Muke | Kazakhstan | AUS | 4 |
| 311180 | 0.436 | Sapundali Local | India | IND | 4 |
| 311745 | 0.447 | Wong Chim | Hong Kong | IND | 4 |
| 311113 | 0.453 | Shui Ya Jien | Hong Kong | IND | 4 |
| 311539 | 0.459 | C.B. II | Japan | AUS | 4 |
| 311423 | 0.460 | IR 58614-B-B-8-2 | Philippines | IND | 4 |
| 310932 | 0.464 | 17-9-4 | Mexico | IND | 4 |
| 310317 | 0.467 | IARI 6626 | India | AUS | 4 |
| 311140 | 0.496 | AKP 4 | India | IND | 4 |
| 311751 | 0.502 | Magoti | Burundi | IND | 4 |
| 311141 | 0.508 | Sornavari | Mali | AUS | 4 |
| 311669 | 0.509 | JP 5 | Pakistan | IND | 4 |
| 311793 | 0.549 | IR64 | Philippines | IND | 4 |
| 311592 | 0.572 | Uz Ros 7-13 | Uzbekistan | AUS | 4 |
| 311606 | 0.581 | Dhan | Nepal | IND | 4 |
| 310630 | 0.598 | Bkn 6987-68-14 | Thailand | IND | 4 |
| 310566 | 0.631 | Iniap 7 | Ecuador | IND | 4 |
| 311153 | 0.636 | IR 2061-214-2-3 | Philippines | IND | 4 |
| 311236 | 0.653 | B805D-MR-16-8-3 | Indonesia | IND | 4 |
| 311654 | 0.669 | Carolino 164 | Chad | AUS | 4 |
| 310615 | 0.794 | Dichroa Alef Uslkij | Kazakhstan | IND | 4 |
| 311242 | 0.341 | Marole | Dominican Republic | IND | 3 |
| 310693 | 0.361 | Bakiella 1 | Sri Lanka | IND | 3 |
| 310481 | 0.364 | Anandi | India | IND | 3 |
| 310503 | 0.376 | Manga Kely 694 | Madagascar | IND | 3 |
| 310598 | 0.396 | Red | Pakistan | IND-AUS-ARO | 3 |
| 310546 | 0.401 | Mahsuri | Malaysia | IND | 3 |
| 311167 | 0.415 | Tainung 45 | Taiwan | IND | 3 |
| 311635 | 0.417 | Amane | Sri Lanka | IND | 3 |
| 310446 | 0.419 | Acheh | Malaysia | IND | 3 |
| 310480 | 0.419 | Djimoron | Guinea | IND | 3 |
| 310415 | 0.421 | Ai Chueh Ta Pai Ku | Taiwan | IND | 3 |
| 311596 | 0.434 | SL 22-620 | Sierra Leone | AUS | 3 |
| 311266 | 0.437 | Co 13 | India | IND | 3 |
| 311765 | 0.438 | Pa Boup | Sierra Leone | AUS | 3 |
| 310519 | 0.452 | Guyane 1 | Chad | IND | 3 |
| 310196 | 0.458 | 81B/25 | Suriname | IND | 3 |
| 311281 | 0.462 | A 152 | Bangladesh | TRJ | 3 |
| 311713 | 0.464 | Sereno | Jamaica | IND | 3 |
| 311188 | 0.473 | Dara | Indonesia | AUS | 3 |
| 311544 | 0.498 | Gallawa | Sri Lanka | AUS | 3 |
| 310351 | 0.512 | Warrangal Culture 1252 | India | IND | 3 |
| 311667 | 0.517 | HKG 98 | Mali | AUS | 3 |
| 311736 | 0.519 | Coppocina | Bulgaria | TRJ | 3 |
| 310348 | 0.522 | C 8429 | Papua New Guinea | TRJ | 3 |
| 310080 | 0.523 | Taichu Mochi 59 | Taiwan | TRJ | 3 |
| 310144 | 0.531 | British Honduras Creole | Belize | TRJ | 3 |
| 311100 | 0.538 | ARC 6578 | India | AUS | 3 |
| 311181 | 0.546 | Tauli | Nepal | AUS | 3 |
| 310779 | 0.546 | GPNO 1106 | Guatemala | TRJ | 3 |
| 311484 | 0.568 | 4595 | China | IND | 3 |
| 311111 | 0.591 | 99216 | India | AUS | 3 |
| 310494 | 0.597 | WW 8/2290 | Netherlands | IND-TRJ-ARO | 3 |
| 311734 | 0.629 | ARC 10633 | India | IND | 3 |
| 310632 | 0.652 | IR 4482-5-3-9-5 | Philippines | IND | 3 |
| 311790 | 0.667 | Wab462-10-3-1 | Cote D'Ivoire | TRJ | 3 |
| 310399 | 0.346 | Doble Carolina | Uruguay | AUS | 2 |
| 310440 | 0.365 | TJ | Guyana | IND | 2 |
| 310715 | 0.406 | Phudugey | Bhutan | AUS | 2 |
| 311656 | 0.468 | Aswina 330 | Bangladesh | AUS | 2 |
| 311269 | 0.489 | Shimla Early | Iraq | IND | 2 |
| 310200 | 0.498 | Chin Chin | Panama | IND | 2 |
| 310338 | 0.521 | Khao Luang | Laos | TRJ | 2 |
| 310023 | 0.528 | RD 218 | Dominican Republic | TRJ | 2 |
| 311123 | 0.529 | 10340 | Italy | IND | 2 |
| 310945 | 0.543 | Dular | India | AUS | 2 |
| 310887 | 0.563 | Buphopa | Myanmar | TRJ-ARO-TEJ | 2 |
| 310901 | 0.579 | Juppa | Nepal | IND | 2 |
| 311435 | 0.589 | Cm1_ Haipong | Vietnam | IND | 2 |
| 311576 | 0.589 | DNJ 121 | Bangladesh | AUS | 2 |
| 310990 | 0.591 | R 67 | Senegal | TRJ | 2 |
| 310238 | 0.614 | R 75 | Senegal | TRJ | 2 |
| 310910 | 0.616 | Tai No 38 | Taiwan | TRJ-IND-AUS-TEJ | 2 |
| 311563 | 0.633 | DNJ 179 | Bangladesh | AUS | 2 |
| 311284 | 0.695 | La Plata Gene F.A. | Argentina | AUS | 2 |
| 311173 | 0.277 | Manga 629 | Madagascar | IND | 1 |
| 310408 | 0.297 | No. Ordem Lista 85 | Brazil | TRJ | 1 |
| 310301 | 0.424 | H57-3-1 | Argentina | TRJ | 1 |
| 311255 | 0.436 | Saraya | Fiji | AUS | 1 |
| 310836 | 0.451 | Gpno 5055 | United States | TRJ | 1 |
| 310381 | 0.458 | NC 1/536 | Pakistan | AUS | 1 |
| 310814 | 0.508 | Grassy | Haiti | TRJ | 1 |
| 310337 | 0.514 | Khao Phoi | Laos | TRJ | 1 |
| 310220 | 0.518 | Safut Khosha | Afghanistan | AUS | 1 |
| 311545 | 0.527 | Ittikulama | Sri Lanka | AUS | 1 |
| 311547 | 0.527 | Karayal | Sri Lanka | AUS | 1 |
| 311383 | 0.530 | Darmali | Nepal | TEJ-TRJ-ARO-AUS | 1 |
| 311668 | 0.531 | Daudzai Field Mix | Pakistan | AUS | 1 |
| 310156 | 0.533 | Sel. No. 388 | Uruguay | TEJ | 1 |
| 310345 | 0.536 | J.P. 5 | Australia | TEJ | 1 |
| 311206 | 0.536 | 79 | Guyana | ARO | 1 |
| 310007 | 0.538 | Karang Serang | Indonesia | TRJ | 1 |
| 310219 | 0.540 | Red Khosha Cerma | Afghanistan | ARO | 1 |
| 310906 | 0.543 | Ardito | Italy | TEJ | 1 |
| 310801 | 0.546 | Tobura | Taiwan | TEJ | 1 |
| 310998 | 0.549 | WC 4443 | Bolivia | TRJ | 1 |
| 310102 | 0.574 | Criollo Chivacoa 2 | Venezuela | TRJ | 1 |
| 310226 | 0.588 | Norin 11 | Japan | TEJ | 1 |
| 311185 | 0.589 | Bombon | Spain | TEJ | 1 |
| 311775 | 0.611 | Thavalu | Sri Lanka | AUS | 1 |
| 311677 | 0.626 | Karabaschak | Bulgaria | TEJ | 1 |
| 310087 | 0.638 | WC 2811 | Micronesia | TRJ | 1 |
| 311573 | 0.654 | DJ 102 | Bangladesh | AUS | 1 |
| 311572 | 0.656 | DJ 24 | Bangladesh | AUS | 1 |
| 310428 | 0.661 | Sipirasikkam | Indonesia | TRJ | 1 |
| 310777 | 0.661 | Wc 3532 | Peru | TRJ | 1 |
| 311644 | 0.664 | P 35 | India | AUS | 1 |
| 311561 | 0.674 | Nang Bang Bentre | Vietnam | AUS | 1 |
| 310958 | 0.701 | 2 | Afghanistan | ARO | 1 |
| 310950 | 0.720 | Nanton No. 131 | Taiwan | TRJ | 1 |
| 311258 | 0.722 | Botika S/R | Zaire | TRJ | 1 |
| 310997 | 0.761 | Lustitano | Portugal | TEJ | 1 |
| 310723 | 0.768 | Wir 3039 | Tajikistan | TEJ | 1 |
| 311016 | 0.769 | IR 238 | Philippines | IND | 1 |
| 311788 | 0.909 | Embrapa 1200 | Brazil | TRJ | 1 |
| 311385 | 0.402 | Kaukkyi Ani | Myanmar | TRJ | 0 |
| 311787 | 0.422 | Krasnodarskij 3352 | Russian Federation | TEJ | 0 |
| 310210 | 0.441 | Krasnodarkij 424 | Russian Federation | TEJ | 0 |
| 310767 | 0.492 | Hb-6-2 | Hungary | TEJ | 0 |
| 310211 | 0.494 | Pergonil 15 | Portugal | TEJ | 0 |
| 310724 | 0.494 | Ak Tokhum | Azerbaijan | ARO | 0 |
| 311779 | 0.497 | WC 10253 | Uncertain | TRJ | 0 |
| 311393 | 0.498 | Celiaj | Azerbaijan | TEJ | 0 |
| 310161 | 0.503 | Shimizu Mochi | Japan | TEJ | 0 |
| 311620 | 0.523 | Romeno | Portugal | TEJ | 0 |
| 310809 | 0.523 | Yong Chal Byo | Korea_ South | TRJ | 0 |
| 311685 | 0.524 | Wir 911 | Russian Federation | TEJ | 0 |
| 310861 | 0.535 | Niwahutaw Mochi | Japan | TEJ | 0 |
| 310747 | 0.544 | Bhim Dhan | Nepal | TRJ-TEJ-ARO | 0 |
| 311532 | 0.547 | Egyptian Wild Type | Turkey | TEJ | 0 |
| 310397 | 0.547 | Chacareiro Uruguay | Uruguay | TEJ | 0 |
| 310965 | 0.561 | Vary Tarva Osla | Portugal | TEJ | 0 |
| 310204 | 0.561 | Italica Carolina | Poland | TEJ | 0 |
| 311794 | 0.574 | M202 | United States | TEJ | 0 |
| 310702 | 0.584 | Jumli dhan | Nepal | TRJ-TEJ-ARO | 0 |
| 311537 | 0.587 | A 5 | Japan | TEJ | 0 |
| 310111 | 0.593 | Bombilla | Spain | TEJ | 0 |
| 310984 | 0.593 | Csornuj | Hungary | TEJ | 0 |
| 310883 | 0.593 | Somewake | Japan | TEJ | 0 |
| 311769 | 0.596 | Pakkali | Philippines | ARO | 0 |
| 311586 | 0.601 | Santhi 990 | Pakistan | ARO | 0 |
| 311642 | 0.602 | Tia Bura | Indonesia | TRJ | 0 |
| 311710 | 0.602 | Lua Chua Chan | Vietnam | TRJ | 0 |
| 310354 | 0.602 | Padi Pohon Batu | Malaysia | TRJ | 0 |
| 310510 | 0.604 | Blue Stick | Fiji | TEJ | 0 |
| 310802 | 0.608 | Tamanishiki | Japan | TEJ | 0 |
| 310588 | 0.611 | Onu B | Zaire | TRJ | 0 |
| 310039 | 0.615 | C 5560 | Thailand | TRJ | 0 |
| 310670 | 0.615 | Kubanets 508 | Russian Federation | TEJ | 0 |
| 311735 | 0.620 | Simpor | Brunei | TRJ | 0 |
| 311497 | 0.635 | Chunjiangzao No. 1 | China | TEJ | 0 |
| 310131 | 0.640 | Secano do Brazil | El Salvador | TRJ | 0 |
| 311600 | 0.656 | Jyanak | Bhutan | TRJ-TEJ-ARO | 0 |
| 311327 | 0.664 | Gasym Hany | Azerbaijan | ARO | 0 |
| 311643 | 0.674 | Padi Tarab Arab | Malaysia | TRJ | 0 |
| 311078 | 0.676 | Gazan | Afghanistan | TEJ | 0 |
| 310645 | 0.681 | Moroberekan | Guinea | TRJ | 0 |
| 311795 | 0.682 | Nipponbare | Japan | TEJ | 0 |
| 310799 | 0.686 | Ragasu | Taiwan | TRJ | 0 |
| 310879 | 0.690 | 6360 | Turkey | TEJ | 0 |
| 310020 | 0.699 | E B Gopher | United States | TRJ | 0 |
| 310045 | 0.743 | Leah | United States | TRJ | 0 |
| 311792 | 0.752 | Cypress | United States | TRJ | 0 |
| 311074 | 0.761 | Mitak | Indonesia | TRJ | 0 |
| 310241 | 0.794 | Uz Rosz M38 | Uzbekistan | TEJ | 0 |
| 310052 | 0.801 | Quinimpol | Philippines | TRJ | 0 |
| CK | 0.472 | Jasmine 85 | United States | IND | 3 |
| CK | 0.945 | Lemont | United States | TRJ | 1 |

* ARO – *aromatic*, AUS – *aus*, IND – *indica*, TEJ – *temperate japonica* and TRJ – *tropical japonica*
